# Parietal maps of visual signals for bodily action planning

**DOI:** 10.1007/s00429-021-02378-6

**Published:** 2021-09-10

**Authors:** Guy A. Orban, Alessia Sepe, Luca Bonini

**Affiliations:** Department of Medicine and Surgery, University of Parma, via Volturno 39/E, 43125 Parma, Italy

**Keywords:** Action observation, Action identity, Social affordance, Posterior parietal cortex, Social interaction

## Abstract

The posterior parietal cortex (PPC) has long been understood as a high-level integrative station for computing motor commands for the body based on sensory (i.e., mostly tactile and visual) input from the outside world. In the last decade, accumulating evidence has shown that the parietal areas not only extract the pragmatic features of manipulable objects, but also subserve sensorimotor processing of others’ actions. A paradigmatic case is that of the anterior intraparietal area (AIP), which encodes the identity of observed manipulative actions that afford potential motor actions the observer could perform in response to them. On these bases, we propose an AIP manipulative action-based template of the general planning functions of the PPC and review existing evidence supporting the extension of this model to other PPC regions and to a wider set of actions: defensive and locomotor actions. In our model, a hallmark of PPC functioning is the processing of information about the physical and social world to encode potential bodily actions appropriate for the current context. We further extend the model to actions performed with man-made objects (e.g., tools) and artifacts, because they become integral parts of the subject’s body schema and motor repertoire. Finally, we conclude that existing evidence supports a generally conserved neural circuitry that transforms integrated sensory signals into the variety of bodily actions that primates are capable of preparing and performing to interact with their physical and social world.

## Introduction

In 1975, Vernon Mouncastle and coworkers summarized the results of a series of single-neuron recordings in the posterior parietal cortex (PPC), stating that “these regions receive afferent signals descriptive of the position and movement of the body in space, and contain a command apparatus for operation of the limbs, hands, and eyes within immediate extrapersonal space. This general command function is exercised in a holistic fashion. It relates to acts aimed at certain behavioral goals and not to the details of muscular contraction during execution” (Mountcastle et al. 1975). More recent evidence provides direct support of this hypothesis for manual actions (Rathelot et al. 2017). However, the rich set of sensory afferents and networks in which the PPC is involved and their relatively conserved organization across mammals (Whitlock 2017) suggest that in addition to directly controlling action execution, a hallmark of the PPC is the integration and exploitation of a variety of sensory signals to specify and select goal-directed actions. Goal-directed actions are conceived as finely orchestrated sequences of body movements aimed to reach a common final goal (Bonini et al. 2013; Orban et al. 2021), thereby enabling individuals to efficiently interact with their physical and social world.

**Fig. 1:**
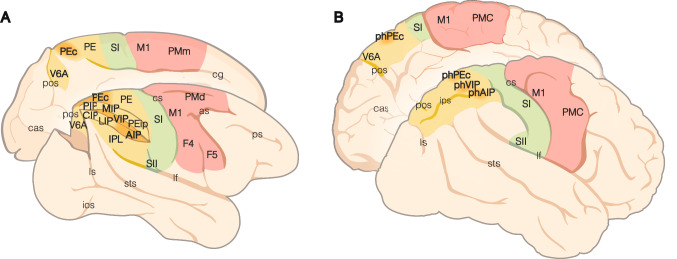
Comparative overview of the monkey and human PPC. **A** Macaque brain areas. **B** Human brain areas. The color code distinguishes frontal motor areas (red), somatosensory cortices (green), and the posterior parietal cortex (yellow; the areas more extensively discussed in this study and their putative human homologues are reported in darker yellow). Abbreviations: *as*, arcuate sulcus; *cas*, calcarine sulcus; *cg*, cingulate gyrus; *cs*, central sulcus; *ios*, inferior occipital sulcus; *ips*, intraparietal sulcus; *lf*, lateral fissure; *ls*, lunate sulcus; *pos*, parieto-occipital sulcus; *ps*, principal sulcus; *sts*, superior temporal sulcus

Neuropsychological studies (Mishkin and Ungerleider 1982; Goodale and Milner 1992) have triggered the highly influential *two visual pathways* hypothesis, in which the dorsal stream projecting to the PPC was deemed to be involved in spatial processing that guides action planning, whereas the ventral stream culminating in inferotemporal regions was thought to be crucial for shape and color processing in the service of object identification. In their concept of the dorsal stream, Milner and Goodale proposed that it includes several maps related to different aspects of the visual space (Milner and Goodale 1993). Accordingly, a series of subsequent studies has parceled the PPC in multiple visually responsive regions (Fig. 1), most notably the anterior intraparietal (AIP), lateral intraparietal (LIP), and caudal intraparietal (CIP) areas, located in the lateral bank of the intraparietal sulcus (IPS); the ventral intraparietal area (VIP) in the fundus of the IPS; the medial intraparietal area (MIP), areas V6Ad/V6Av in its medial bank; and area PEc on the crown of the hemispheres (Lewis and Van Essen 2000a; Gamberini et al. 2020). Single-cell studies have demonstrated that these visual areas play a major role in the process of selection and decision among different behavioral alternatives; for example, area AIP receives information about graspable objects (Murata et al. 2000) and selects the most suitable affordances to be turned into the appropriate hand shape for grasping them (Tunik et al. 2005; Schaffelhofer and Scherberger 2016), and area LIP/parietal reach region (PRR) encode decisions about reaching toward alternative targets with the eye or with the arm, respectively (Snyder et al. 2000; Huk et al. 2017). An influential view of this organization proposed the existence of effector-centered *intentional maps* (Andersen and Buneo 2002): AIP for hand movement, LIP for eye gaze, and PRR/MIP/V6A for arm or arm and hand movement. It is unclear, however, whether and how this view can be extended to the full behavioral repertoire of primates and to other PPC regions, because several recent findings suggest a prominent role of mixed coding of effector-related signals in the PPC (Lehmann and Scherberger 2013; Zhang et al. 2017; Hadjidimitrakis et al. 2019; Diomedi et al. 2020).

Single-neuron recording studies have shown that cells in most of these nodes of the PPC not only become active during the observation of a target object or location, action planning, and execution, but can also discharge during the observation of others’ actions: manual actions in AIP (Pani et al. 2014; Maeda et al. 2015; Lanzilotto et al. 2019, 2020), reaching–grasping actions in V6A (Breveglieri et al. 2019), and even eye gaze shifts in area LIP (Shepherd et al. 2009). Importantly, all these studies have shown clearly that although neurons can code both self and others’ actions, they exhibit different discharge levels and dynamics. For example, neurons in area AIP that discharge during grasping execution can exhibit suppressed discharge during action observation (Lanzilotto et al. 2019), as has previously been demonstrated in many premotor regions (Jerjian et al. 2020; Ferroni et al. 2021). Furthermore, decoding analyses carried out in other cortical areas that host neurons encoding both self and others’ action have directly demonstrated the possibility of reliably discriminating the agent (self or other) of an ongoing action based solely on neuronal activity readout (Livi et al. 2019), thereby demonstrating that primates’ brains clearly distinguish between the subject’s own actions and those performed by others.

We recently proposed that both signals related to manipulable objects and those related to others’ actions may provide support for a unique and fundamental function of the PPC: generating a variety of sensory-driven action and interaction opportunities that encompasses object (Cisek 2007; Maranesi et al. 2014; Pezzulo and Cisek 2016) and social (Orban et al. 2021) affordances. These concepts fit well with the accumulating evidence favoring the existence of “action-fields” surrounding the body (Bufacchi and Iannetti 2018), which match the variety of behavioral actions that aim to establish or avoid contact with objects, other individuals, or even specific body parts of the subject. Our proposal here is to extend the framework originally conceived for object-directed manipulative actions to the entire variety of action opportunities offered to the subject by its physical and social environment.

Unfortunately, the opportunities to investigate the large variety of behavioral actions of both the human and nonhuman primate repertoire have thus far been limited by the constraints imposed by the techniques available for the recording of whole-brain or single-neuron activity. In fact, the overwhelming majority of existing studies have focused only on those movements that can be explored in a head-fixed monkey sitting in a primate chair or in human subjects lying still inside the bore of an MRI scanner. Clearly, investigations of highly restrained subjects can cover only a minimal part of the large and refined behavioral repertoire of monkeys and humans. However, in an observation (rather than execution) mode, it is also possible to investigate the brain coding of actions performed with the foot or the mouth (Jastorff et al. 2010), whole-body actions such as locomotion or climbing (Abdollahi et al. 2013), skin displacing (Ferri et al. 2015b) or vocal communication (Corbo and Orban 2017), at least in terms of the coarse underlying neural mechanisms. Of course, the organization, planning and control of these action classes at the single-neuron level remains unresolved, because fMRI approaches, used in the aforementioned human action observation studies, cannot offer reliable measures of neural selectivity (Sawamura et al. 2006; Dubois et al. 2015). However, a few single-neuron studies in both monkeys (Lanzilotto et al. 2019, 2020) and humans (Aflalo et al. 2020) have provided strong mechanistic support for a novel picture of action planning, at least for manipulative actions (Orban et al. 2021). We showed that during monkeys’ observation of videos depicting a variety of manipulative actions, neurons in area AIP exhibited selectivity for the observed-action identity (e.g., dropping, pushing, grasping, etc.), in addition to responding during grasping execution in the dark (Lanzilotto et al. 2020). These findings, paralleled by remarkably similar properties reported for neurons of the putative human homolog of AIP (phAIP) tested with the same set of videos (Aflalo et al. 2020), prompted us to suggest that AIP provides not only object affordances, but also social affordances, thereby constituting a potentially unitary mechanism for the planning of manipulative actions (Orban et al. 2021). Here, we propose that the available evidence about other PPC regions justifies an extension of this model to a larger territory of the PPC and a wider set of sensory inputs, enabling the exploitation of sensory signals for planning potential behavioral responses to both the physical and social environment.

In what follows, we thus start with our recent proposal of social affordances in AIP to (1) derive the set of signals used for manipulative-action planning in AIP; (2) propose an AIP-based template model of the possible broad functioning of the PPC and apply this template model to PPC regions other than AIP and to two additional action classes: defensive and locomotion actions; and (3) further extend this model to actions performed with man-made objects (e.g., tools).

## Visual and haptic signals for manipulative-action planning

### Object-related signals and motor affordances

Classical models of object affordance maintain that the properties of objects constituting potential targets of the subject’s manipulative actions are processed along the dorsal stream, with area AIP and its projections to area F5 playing a pivotal role in turning the visual features of the object into the appropriate hand shape for interacting with it (Jeannerod et al. 1995). Specifically, the 3D shape of the object, its size, and its orientation (Murata et al. 2000) constitute the most frequently tested properties in neurophysiological experiments in monkeys (Schaffelhofer and Scherberger 2016) and in noninvasive human studies (Tunik et al. 2005). A combined intracortical microstimulation and fMRI study in the monkey (Premereur et al. 2015) showed that the caudal part of AIP exhibit connectivity with both the caudal regions of the lateral IPS (LIP and CIP) and the caudal STS region known to code 3D shape from motion (Mysore et al. 2010), whereas a more rostral part of AIP is linked with SII, premotor area F5 and the intermediate part of the lateral and medial IPS. Thus, combined visual, motion, and somatosensory signals converge in AIP in a caudo-rostral visual-to-motor gradient (see also Lanzilotto et al. 2019), contributing to a multimodal specification of *how* to interact with an object. However, color, glossiness and 3D texture, as well as other visual signals about material properties of objects, provide important clues about their behavioral relevance. For example, the ripeness or palatability of a fruit suggests *what* to do with it (Bruni et al. 2017; Maranesi et al. 2019). These properties concern the physical appearance of objects, thereby conveying “semantic” information in addition to the pragmatic description. This semantic information reaches AIP not only via the well-established intraparietal (Nakamura et al. 2001) and inferotemporal-inferior parietal routes (Borra et al. 2008; Nelissen et al. 2011), but also through nonvisual pathways arising from the ventrolateral prefrontal cortex (Bruni et al. 2015) and SII (Borra et al. 2008; Lanzilotto et al. 2019). Indeed, in addition to vision operating before the contact between hand and object, several properties of target objects can be revealed directly from their haptic or visuo-tactile exploration (Reed et al. 2004; Bruni et al. 2017), and the convergence of this information in area AIP can be important for supporting the planning and execution of action with reference to the behavioral meaning of the target object. Here, we refer to the sets of pragmatic and semantic visuo-tactile information subserving manipulative-action planning as “sensory features of the environment”.

### Others’ observed actions and social affordances

In addition to the above-described sensory features of the environment, recent single-neuron studies have revealed selectivity for observed manipulative actions (OMAs) in visuomotor neurons of monkey AIP (Lanzilotto et al. 2019) and in human phAIP neurons (Aflalo et al. 2020). These studies have shown that specific observed-action exemplars depicted in brief videos can be decoded from the responses of AIP and phAIP neurons even across viewpoints (e.g., lateral/frontal), supporting the idea that neuronal populations in these areas encode OMA identity. In particular, we proposed (Orban et al. 2021) that OMA identity is computed from two distinct STS signals reaching AIP, the first concerning body shape changes and originating in areas PGa/IPa (Vangeneugden et al. 2009), and the second concerning the attainment of the goal by others’ hand–object interaction provided by area TEa (Perrett et al. 1989). Therefore, we suggest that, as previously hypothesized for objects (Cisek 2007), the encoding of specific observed actions’ identity by AIP neurons endowed with motor properties can lead to the specification and selection of the potential motor actions required to interact with the observed agent, thereby extending the concept of affordances from objects to others’ actions. Accordingly, we designate as “social affordances” the variety of competing potential actions from which an observer can select the most suitable one for interacting with the observed agent.

The social-affordance concept can advance the existing literature on mirror neurons (Rizzolatti et al. 2014), because it does not necessarily imply a direct match between the observed action and its motor representation in the observer’s brain. Such flexibility has been firmly established for objects. For example, a cup of coffee can afford a variety of alternative actions, from grasping it by the handle with a precision grip or with a hook grip (by inserting the index finger in the handle cavity) if it is full of hot coffee; grasping it from the top or the side with a whole-hand prehension if it is full of liquid that does not appear to be hot; or putting the fingers inside and on the side of the cup if it is empty and clean and the objective is simply to rapidly move it away. Multiple affordances are simultaneously available in our motor system (Borghi and Riggio 2015), and they undergo a process of progressive specification and selection along the AIP-F5 pathway, which ultimately recruits the motor cortex (Schaffelhofer and Scherberger 2016) to finally perform the action that appears to be more relevant in the current context (Baumann et al. 2009). We could kick the cup or grasping it with the mouth, but these are certainly not the most readily available affordances for a healthy human in normal situations, because despite the motor system’s considerable flexibility, some alternatives are more plausible in view of the subject’s behavioral repertoire.

Similarly, we propose that the very same process operates when the sensory input concerns not an inanimate object, but the action performed by a conspecific. Indeed, observed actions can afford multiple (independent, mutual or competitive) behavioral reactions. For example, if someone pushes an object toward us, we may be induced to grasp it, push it back to the partner, throwing it away, etc., depending on the nature of the object (a certain palatable or disgusting food) and on our internal (e.g., motivational) state. In this case, it is also clear that some visuomotor correspondences between observed actions and the afforded behavioral response are stronger and more readily accessible than others: one’s own actions afforded by others’ observed actions are most frequently those belonging to the same action class, but certainly not necessarily to the very same action. In this view, it is useful to note that strictly congruent selectivity for executed and observed grip type (e.g., precision grip, finger prehension, whole-hand prehension, etc.) in ventral and dorsal premotor neurons with mirror-like properties occurs at the chance level (Papadourakis and Raos 2019). In addition, in the mesial premotor cortex (Livi et al. 2019) and parietal area V6A, mirror-like neurons almost completely lack visual selectivity for the observed grip type (Breveglieri et al. 2019). Furthermore, AIP neurons that respond during active grasping in the dark can be visually tuned to any of the other’s action exemplars tested, not just to “grasping” (Lanzilotto et al. 2019). Thus, a hallmark feature of visuomotor neurons encoding others’ manual actions appears to be the presence of a genuine motor response rather than a match between the visual and motor selectivity, supporting the idea that, as proposed for observed objects, the sight of others’ actions flexibly recruits multiple behavioral responses in the observer’s brain, depending on the observed action in the current context.

### Sensory and motor signals related to the execution of manipulative actions

Two other important signals contribute to the planning and monitoring of a manipulative action during its unfolding in the PPC, particularly in area AIP: (1) visual feedback about the movement of the subject’s own hand and the target (Pani et al. 2014; Maeda et al. 2015; Lanzilotto et al. 2019), and (2) proprioceptive signals carrying information about the dynamic state of the forelimb (Gardner et al. 2007).

Concerning the visual feedback about the subject’s own hand, pioneering studies of AIP single-neuron activity have revealed the presence of cells, called “visual dominant non-object type” neurons that discharge during grasping in the light but not in the dark and exhibit no response to the visual presentation of graspable objects. Interestingly, more recent evidence shows that in AIP, a small fraction (≈ 15%) of neurons showing suppressed discharge during the visual presentation of manipulative-action videos exhibited facilitated modulation of their discharge during grasping in the light but not in the dark (Lanzilotto et al. 2019), suggesting that they may represent the visual feedback about the subject’s own action, thereby contributing to own action monitoring (Sakata et al. 1995). Indeed, AIP has been causally linked with visually guided control of manual actions (Gallese et al. 1994), and own hand visual feedback seems to be particularly relevant for this function. Own hand visual feedback is encoded by area F5 (Maranesi et al. 2015) in addition to AIP, and constitutes the next step of the parieto-frontal circuit for the visually guided control of grasping (Fogassi et al. 2001).

Besides visual information, AIP also receives strong somatosensory afferents (Lewis and Van Essen 2000b), especially in its more rostral part (Lanzilotto et al. 2019). These somatosensory inputs, especially afferents from SII (Borra et al. 2008), may participate in the haptic identification of the target object, a particularly refined skill in Old World monkeys, apes and humans relative to New World monkeys (Kaas and Stepniewska 2016), whose parietal cortex exhibits a simpler organization (Padberg et al. 2005; Burman et al. 2015). Therefore, the haptic processing of objects likely plays a major role in contributing to the planning and monitoring of the subject’s own manual actions, depending on the nature, value and behavioral relevance of the manipulated target. In addition, consistent projections from the intraparietal area PE (PEip) and the parietal operculum in macaques (Borra et al. 2008; Lanzilotto et al. 2019) may provide preprocessed proprioceptive and kinesthetic feedback about the ongoing action. Based on these latter studies, which have shown that visual afferents prevail in the caudal part of AIP whereas projections to the premotor cortex and proprioceptive/somatosensory afferents predominate in its rostral part, it may be suggested that the subject’s own action planning and monitoring proceeds from the caudal to the rostral sectors of AIP: from the characterization and selection of potential physical and/or social targets in the caudal part to the ongoing control and monitoring of the selected motor plan in the rostral one. Of course, AIP works in concert with the ventral premotor areas (F5 and F2vr), in line with their tight anatomical (Borra et al. 2008; Nelissen et al. 2011; Lanzilotto et al. 2019) and functional (Schaffelhofer and Scherberger 2016) coupling during manual-action execution.

### Summing up: object and action signals for manipulative-action planning

The view so far proposed concerning manipulative actions fits well with the influential *affordance competition hypothesis* (Cisek 2007), but expands it beyond visual information about 3D physical properties of objects and their behavioral relevance to include observed actions as well. Notably, these signals converge up to the single-neuron level in the premotor cortex (Maranesi et al. 2012; Bonini et al. 2014) and very likely in AIP as well (Ferroni et al. 2021), although a direct demonstration of the latter integration is still lacking. In addition, both area F5 and AIP receive consistent afferents, directly and indirectly, from the prefrontal and presupplementary motor cortices (Bruni et al. 2018; Lanzilotto et al. 2019; Albertini et al. 2020) as well as from the basal ganglia (Gerbella et al. 2016; Borra et al. 2021), which also contribute to the evaluation and selection of action plans based on the external and internal state and goals of the subject.

**Fig. 2:**
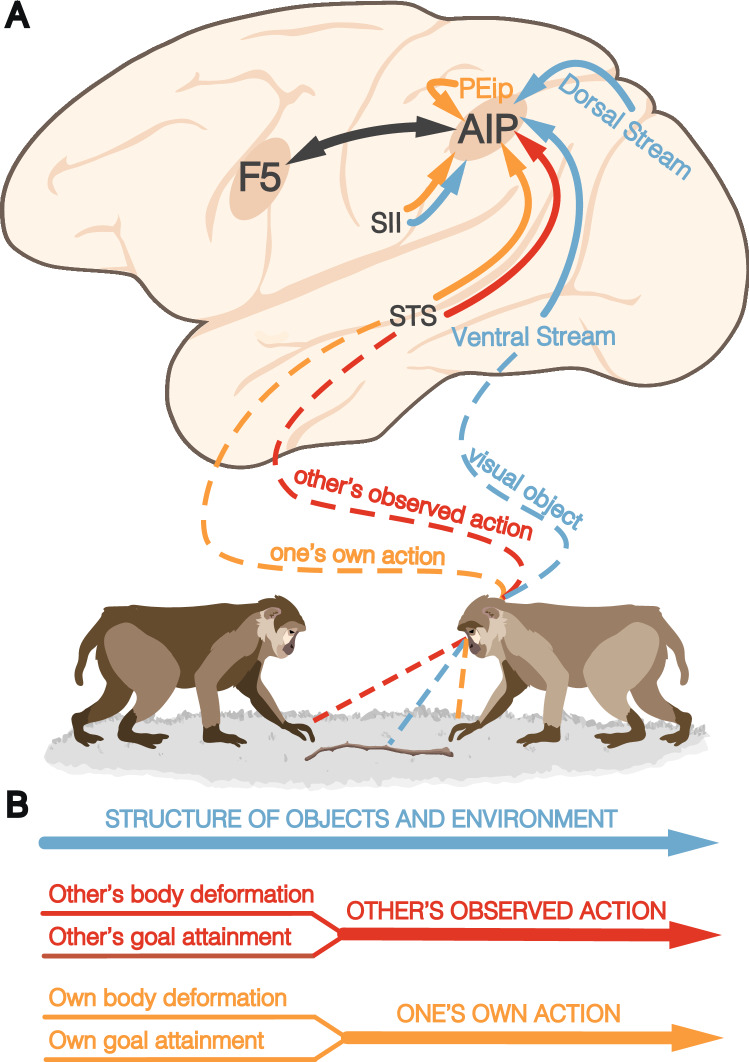
Convergence of visual objects, observed actions, and own hand visual and proprioceptive signals in PPC territories devoted to manipulative actions. **A** Own action planning benefits from (1) motor affordances provided by the 3D structure of objects and the environment (blue), (2) social affordances provided by others’ observed actions (red), (3) own actions’ visual and somatosensory/proprioceptive feedback from the subject’s body (orange). Distinct anatomo-functional visual components of observed actions can be identified in the goal-attainment signals (rostral STS) and body movement signals (middle STS) concerning the observed actions of others, which are paralleled by the analysis of visual/proprioceptive feedback from the subject’s own actions (conveyed by the STS, PEip, SII, and F5, particularly concerning haptic information about the manipulated object). **B** Schematic view of the different signals that contribute to the encoding of the physical properties of objects and the environment, the subject’s own actions, and others’ actions. *PEip* intraparietal area PE, *SII* secondary somatosensory cortex, *STS* superior temporal sulcus

In conclusion, the planning of manipulative actions involves three sets of signals (Fig. 2): (1) affordances of physical objects and surfaces of the environment, conveyed mainly by visual signals but, as reported in AIP, often complemented by tactile and proprioceptive information, (2) social affordances provided by observed actions of others, and (3) multimodal signals about the subject’s own actions and the current state of the effectors. We propose that these three sets of signals (Fig. 2B) may characterize the different PPC modules involved in the planning of the variety of action classes constituting the behavioral repertoire of primates (Orban et al. 2021). The subsequent sections will thus explore this possibility for two main action classes: defensive and locomotor actions.

## Visuo-tactile integrations contributing to defensive-action planning

Pioneering neurophysiological studies in the monkey have revealed that a network of tightly interconnected parietal (Duhamel et al. 1998), ventral premotor (Gentilucci et al. 1983; Graziano et al. 1994; Fogassi et al. 1996), and basal ganglia (Graziano and Gross 1993) regions host somatosensory and visual neurons deemed to play a fundamental pragmatic role, that is, to guide hand actions in the peripersonal space (Graziano et al. 1994; Rizzolatti and Matelli 2003; Gharbawie et al. 2011). Studies have subsequently produced causal evidence that the artificial activation (intracortical microstimulation or local drug injections) of both the parietal (Thier and Andersen 1998; Cooke et al. 2003; Stepniewska et al. 2005) and premotor (Graziano et al. 2002; Cooke and Graziano 2004) nodes of this circuit cause a variety of defensive reactions. Thus, the most recent proposals emphasize the relevance of this bimodal, or even multimodal (Graziano et al. 1999), integration for the emergence of graded fields of behaviorally relevant actions aiming to promote—or most often, avoid—the contact between objects and the body (Bufacchi and Iannetti 2018).

Here, we will apply the framework previously proposed for manipulative actions (see Fig. 2) to the class of actions specifically aimed at preventing or avoiding the contact of objects with the body (defensive actions), considering again the case of other agents—in addition to that of physical objects—as a potential source of threats and affordances for one’s own defensive-action planning and execution.

### Parrying and avoiding objects directed toward the body

Multimodal integration in area VIP has long been recognized as a crucial step in the emergence of action organization and space perception (Duhamel et al. 1998; Schlack et al. 2005), particularly insofar as defensive actions are concerned. The visual, tactile, and auditory convergence region of VIP is devoted mainly to representing the face, but according to fMRI studies (Guipponi et al. 2013), this is only part of the visual motion-sensitive region lying in the fundus of the intraparietal sulcus (Lewis and Van Essen 2000a). By extrapolating functional markers from monkey single-neuron studies and combining tactile, visual, and auditory stimuli, researchers initially localized the putative human homolog of VIP (phVIP) in the fundus of the IPS (Bremmer et al. 2001). However, subsequent fMRI studies based on different sets of stimuli, validated in the monkey (Cottereau et al. 2017), have suggested a more dorsal location of phVIP (Cardin and Smith 2010), which fits well with the expansion of the IPL in humans (Grefkes and Fink 2005; Mantini et al. 2013).

Importantly, electrical intracortical stimulation (ICMS) of area VIP (Thier and Andersen 1998; Cooke et al. 2003) causes eye closure, grimacing, head withdrawal, shoulder elevation, and arm protective movements, which correspond globally to defensive actions. In fact, very similar reactions can be evoked by natural aversive stimuli, such as air puffs of controlled intensity applied to specific body regions (Cooke and Graziano 2003). Further support for the “defensive” interpretation derives from remarkably similar results obtained from the application of ICMS to the main target of anatomical projections of VIP (Luppino et al. 1999), that is, the ventral premotor area F4. In this latter region, long-train ICMS elicited reactions remarkably similar to those following VIP stimulation (Graziano et al. 2002), whereas chemical stimulation (bicuculline) and inactivation (muscimol) of area F4 (Cooke and Graziano 2004) increased and decreased the monkey’s reactions to aversive stimuli, respectively. Thus, there is clear causal evidence that VIP, in concert with area F4, plays a crucial role in the planning and control of defensive reactions to visual stimuli approaching specific body parts, particularly the upper body and the face.

What are the functional properties of VIP neurons, and where do the sensory signals converging in VIP come from? Duhamel and coworkers (Duhamel et al. 1998) reported that the majority (70%) of VIP neurons are bimodal visuo-tactile neurons. Moreover, the location of the visual receptive field (RF) matched that of the tactile RF, with the foveal region grossly corresponding to the nose/mouth and the visual and tactile RF increasing as one moves away from the personal and peripersonal space linked to the snout. Most neurons (85%) were motion selective, with matched direction selectivity in the tactile and visual modes. In some cases, bimodal response patterns were particularly complementary: cells responding to objects moving in depth toward the monkey exhibited increased activity to tactile stimulation onset, whereas cells responding to objects moving away from the monkey exhibited increased activity to the end of the tactile stimulus application. This match between tactile and visual modalities was facilitated by the integration of eye position and visual signals, with the individual receptive fields of VIP neurons being organized along a continuum, from eye to head coordinates (Duhamel et al. 1997), and becoming further independent from retino-centric coordinates when traveling to area F4 (Fogassi et al. 1996).

Concerning tactile input, areas in the medial wall of the IPS (e.g., MIP and PE) likely represent an important source of somatosensory information for VIP, in addition to primary somatosensory areas (Seltzer and Pandya 1986; Lewis and Van Essen 2000b). Concerning visual input, VIP receives direct projections from area MT (Maunsell and van Essen 1983), which may account for its motion selectivity. Interestingly, visually responsive VIP neurons exhibit a considerable selectivity for visual disparity (Bremmer et al. 2013), demonstrating a remarkable overrepresentation of very negative disparities, which corresponds to a preferential coding of the nearby space (< 30 cm from the head), in line with earlier observations (Colby et al. 1993). Among the visuo-tactile VIP neurons, of particular interest are those selective for looming visual stimuli. Looming stimuli induce avoidance reactions in rhesus monkeys (Maier et al. 2004) in early life (Schiff et al. 1962), and the same effects have been observed in humans beginning with week two of postnatal age (Ball and Tronick 1971). These reactions persist after the removal of the primary visual cortex, when the subject is hypothetically blind (King and Cowey 1992), suggesting they do not critically depend on conscious perceptual processing of the stimuli. Instead, these avoidance reactions in the absence of the primary visual cortex require intact visual input originating from nonprimary visual brain regions, such as areas MT/MST (Cléry et al. 2017), which are in turn the targets of direct and indirect projections not only from area V1, but also from visually responsive subcortical regions, such as the superior colliculus (Rodman et al. 1990; Lyon et al. 2010; Berman and Wurtz 2010).

These visuo-tactile afferents and the deriving functional properties appear to allow the VIP–F4 circuit to robustly fulfill a fundamental, automatic defensive function, triggering potential motor actions of the subject even in the presence of only a partial sensory description of the looming stimulus. This mechanism is similar in terms of anatomical and functional organization to that previously described for the object affordance of manipulative actions; for parrying and avoiding objects (especially the looming ones), as well as for reaching and grasping them, competing motor actions are automatically recruited to preserve the integrity of the body from external threats.

### When the threat is the other: social affordances for defensive actions

Most of the existing literature has focused on the study of peripersonal space using inanimate objects as stimuli to probe neuronal and brain responses. This contrasts with the obvious evidence that other agents can be particularly threatening stimuli because of their capacity to move, which makes them highly unpredictable and thus requires the subject to promptly access and activate potential motor actions to, for example, deal with a possible attack. Attacks and fights are indeed very frequently in many nonhuman primate species, particularly macaques (Thierry et al. 2004), and may have contributed to the evolution of sophisticated, defensive mechanisms that treat social stimuli as particularly salient.

Since the very first descriptions of peripersonal neurons, their testing has implied that the experimenter stood in front of a head-fixed animal and moved his/her hands, which held sticks or objects of different types, toward the monkey’s body (Gentilucci et al. 1988). In subsequent studies of the properties of both premotor (Maranesi et al. 2012) and inferior parietal lobule (Rozzi et al. 2008; Ishida et al. 2010) neurons, visual responses were preliminarily tested simply by the experimenter waving his/her hand toward the animal, that is, with no other stimulus than the experimenter’s intransitive gesture. In all these cases, peripersonal neurons typically respond, indicating that they can be activated by moving stimuli constituted by other agents’ movements. In line with this evidence, the hand-related blink reflex, which occurs when a threat is brought close to a human subject’s face by the subject’s own stimulated hand, has been observed also when another person’s hand brings the threat close to the subjects’ face, regardless of the (egocentric or allocentric) viewing perspective (Fossataro et al. 2016), suggesting that social interactions shape the perception of threat and defensive responses. Several human studies have supported the existence of a variety of possible social modulations of the multisensory-motor representation of peripersonal space (Heed et al. 2010; Teneggi et al. 2013; Fossataro et al. 2016). Although, to our knowledge, no study has ever directly compared peripersonal neurons’ response to physical and social stimuli in primates, these observations indicate that social affordances likely exist for defensive, in addition to manipulative, actions. Furthermore, a monkey single-neuron study testing the response of peripersonal neurons to stimuli moved toward the subject’s own or another’s body provided evidence for a possible shared coding of self and others’ defensive actions in a body-centered reference frame (Ishida et al. 2010); this may represent an automatic, vicarious response that increases the salience of a given body-centered action even if it is not directly triggered by a physical stimulus but, instead, by the sight of a peer experiencing the same threat. A similar mechanism may be especially relevant if the peer is very close to the subject, as suggested by the human data reviewed above (Fossataro et al. 2016) and by fMRI studies showing that observing interactions with other passive conspecifics activates the phVIP (Ferri et al. 2015b).

The anatomical connectivity underlying social affordances for defensive actions likely depends on two main visual pathways reaching area VIP. The first one, concerning others’ body movement, likely depends on the projections from the STS complex formed by areas IPa/PGa (Lewis and Van Essen 2000b), whereas the second one specifies the relationship between the motion of the conspecific and the location of the threatening object, which, depending on the viewpoint, may require 2D or 3D (motion parallax) processing. Although the neural substrates of relative motion processing have received little attention, the MT complex might provide such signals (Tanaka et al. 1993; Nadler et al. 2009; Kim et al. 2015). Of course, the observation of others’ defensive actions is a relatively infrequent but highly salient stimulus, and can occur when conspecifics do not face each other. Indeed, social affordances for defensive actions are particularly important when a group of conspecifics faces a common threat, as on a battlefield, where observing the defensive actions of others who are more directly exposed or better able to perceive a threat may automatically retrieve and specify the internal representation of defensive actions in the observer’s brain. Another situation that triggers defensive actions is fighting, in which subjects face overtly offensive actions directed to them by others; in this case, however, the mechanisms triggering the subject’s own potential defensive actions are likely the same as those recruited by inanimate objects threatening the subject’s own body, as described above. In fact, when something threatens to harm the body, it is not so relevant whether it is an object or another subject; in both cases, there is an urgent need to enhance and select motor actions aimed at protecting the body part of the subject most likely to be affected by the threat.

### Feedback signals about the execution of defensive actions

The properties of bi- or multimodal neurons thus far reviewed indicate that the convergence of somatosensory and visual/auditory signals serves to anchor sensory representation of looming/receding stimuli to a body-centered reference frame, which is necessary primarily for the preparation of defensive actions, not for the representation of space. The goal of defensive actions essentially consists in preventing the impact of objects with the body or minimizing its effects. Indeed, stimuli approaching at higher speeds cause earlier and stronger firing of peripersonal neurons (Fogassi et al. 1996), because earlier and faster preparation for a defensive response is required in these cases. This is in line with the evidence of overt defensive movement triggered by area F4 (Graziano et al. 2002) and VIP (Cooke et al. 2003) electrical stimulation. In this view, visual signals related to the subject’s own defensive actions likely play a more negligible role, because defensive actions are typically more ballistic and require less refined control than actions involving, for example, skilled finger movements to interact with objects or other subjects. Yet, signals related to visual motion in depth, which can originate from both monocular (expansion/contraction) and binocular processes deriving from the subject’s own movement in the environment, are conveyed by area MT (Czuba et al. 2014) and typically distinguish between stimuli directed to and those directed away from the head, playing a plausible role in complementing tactile information for a successful, active avoidance of threatening stimuli.

A bulk of data also suggest that visceromotor feedback signals make an important contribution to the individual’s autonomic state associated with defensive actions. These feedback signals work in parallel with the properties of the parieto-frontal system thus far reviewed and contribute to assigning a valence or relevance to the subject’s own potential defensive actions depending on the available sensory and contextual information (Ferri et al. 2015a; Dureux et al. 2021). Indeed, viewing threatening social stimuli per se causes the activation of subcortical structures such as the amygdala, periaqueductal gray, and hypothalamus (Pichon et al. 2012) as well as the parieto-premotor circuits; these subcortical brain structures can promote an arousal and emotional state in the subject that is optimal for appropriately facing physical or social threats. Even in this case, however, the impact of the subject’s own emotional and physiological state on the functioning of the parieto-frontal neural systems underlying the selection and execution of defensive motor plans has yet to be investigated.

### Summing up: object and action signals for defensive action planning

**Fig. 3:**
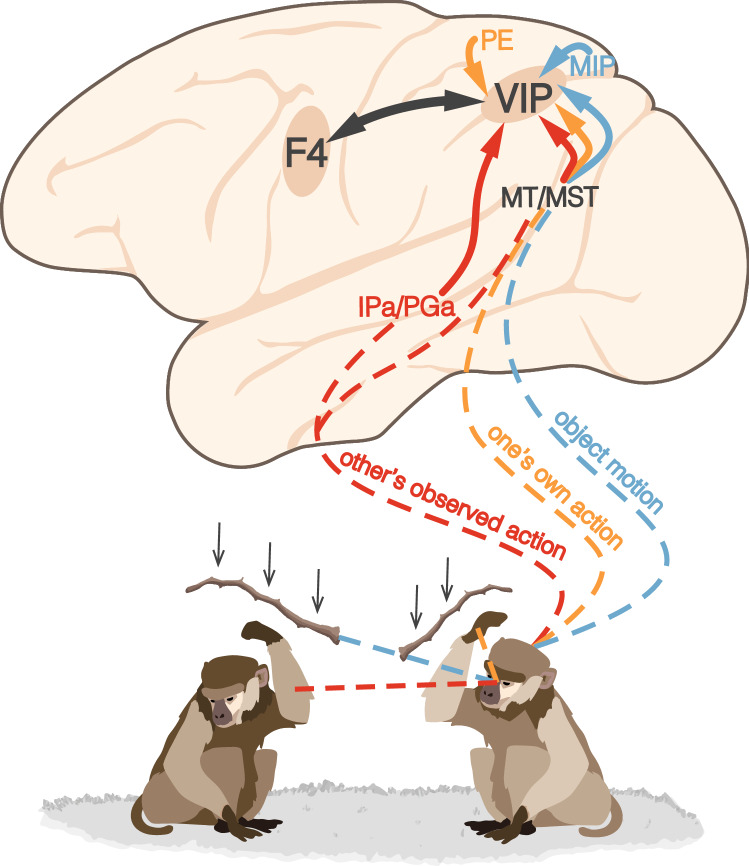
Parieto-frontal circuit for defensive actions. Convergence of the visual object’s motion (blue), observed actions (red), and own hand visual and proprioceptive signals (orange) in the PPC territory devoted to defensive actions, namely the VIP, which is directly linked with ventral premotor area F4

We have proposed that, as for manipulative actions mostly encoded in AIP, the subject’s own defensive actions are automatically afforded by the sight of objects moving toward the subject’s body and depend largely on area VIP (Fig. 3). The functional difference between these two areas is also supported by the differences in local and long-range connectivity (Luppino et al. 1999), as was elegantly summarized by a recent hierarchical cluster analysis based on virtually all the previously published retrograde-tracing studies (Caminiti et al. 2017). The results highlight, on one side, the tight relationship of AIP with somatomotor areas of the lateral inferior parietal convexity (Rozzi et al. 2006; Borra et al. 2008; Gamberini et al. 2009; Bakola et al. 2017; Lanzilotto et al. 2019) and the ventral premotor cortex (Borra et al. 2008; Lanzilotto et al. 2019), and on the other side, the larger variety of connectional pathways of VIP with brain areas related to visual, somatosensory, vestibular and multisensory processes (Lewis and Van Essen 2000b), including prefrontal cortical areas (Caminiti et al. 2017). These connectivity profiles fit well with the more specific role of AIP as a hub for information concerning observed and executed manipulative actions, particularly its more caudal part (Lanzilotto et al. 2019), whereas area VIP may exploit the variety of convergent multisensory information regarding the face/head region to plan and execute a large and heterogeneous set of defensive actions. This is coherent with the evidence that both inanimate physical objects and others’ actions approaching the subject’s body may act as equally optimal stimuli affording a variety of defensive actions accompanied by an appropriate emotional/arousal state.

Yet, despite the analogies with the manipulative-action class, the detailed encoding of defensive-action identity may seem less crucial, because the subject’s need to promptly react to a threatening stimulus in order to protect his/her own body renders the nature of the stimulus—whether it is a physical object or another subject—rather irrelevant. In fact, the importance of social affordances for defensive actions may depend very heavily on the social context and likely applies more to groups of subjects than pairs of conspecifics, in which subjects may be processed more similarly to any physical object. An important limitation of this literature, which is due mainly to technical limitations, is the lack of studies on active avoidance in ecological conditions. Indeed, many defensive actions depend on the possibility of escaping from the threat, which is obviously impossible when the subject is sitting on a primate chair or lying in the bore of an MRI scanner. Some attempt have been made to test the neural substrates of the passive and active avoidance of impending obstacles by an observer in a wide-field virtual reality environment, suggesting that active avoidance involves slightly different and more specific activations within a network of areas largely shared by the passive view of egomotion (Huang et al. 2015). These findings indicate that VIP may provide input to a larger brain network (Serra et al. 2019) depending on the context, possibly playing a role in a larger set of avoidance behaviors, including fleeing. Although the motor sequences required for fleeing can be remarkably similar to those required for locomotion, the fundamental distinction resides in the final goal of these two action classes: to move away from a threat and to move toward a desirable target out of the arm’s reach, respectively. Critically, these studies will require necessarily unconstrained settings and freely-moving conditions, as well as the possibility to manipulate social context (group size) and the potential available targets while testing neuronal activity.

## Visual, vestibular and bodily signals for locomotor actions

We certainly live in a world full of action choices (Cisek and Kalaska 2010), but very often in human and nonhuman primates’ daily life, many visible targets—such as a cozy place, a desirable object, a partner or another animal—are out of immediate reach and require the subject to move his/her own body in space to approach them for a variety of purposes, thus making locomotor actions directed toward physical and social targets a crucial component of primates’ behavioral repertoire.

Locomotor actions generally depend on the typically rhythmic motor behaviors that enable humans and other animals to move in a medium, such as water, land, or vertical structures via behaviors such as swimming, walking, or climbing, respectively. Although the spinal circuitries subserving locomotion are thought to be rather mechanistic and have been described with considerable precision (Kiehn 2016; Minassian et al. 2017), how animals decide and control where and how to move to forage or escape a predator requires planning and control functions exerted by supraspinal brain regions, particularly the PPC (Drew and Marigold 2015). These planning functions may be even more relevant for nonrhythmic locomotor actions, such as jumping or diving.

Before addressing the issue of the variety of signals used by the brain to plan and control the supraspinal aspects of *goal-directed* locomotor actions, it is important to make a preliminary distinction. Of course, locomotion is intimately linked with, and instrumental to, navigation. Human studies using virtual reality have suggested that a facilitation effect on walking-related actions can be obtained at the sight of targets located up to 40 m from the subject (Di Marco et al. 2019), but this does not obviously entail that the full locomotor-action chain has to be planned at the sight of the distant target. Indeed, we propose that whereas navigation strictly concerns the localization of the subject relative to external references and possible target goals at any distance in the environment (i.e., “where to go”), locomotion is more closely linked with the motor actions required to achieve a visible target in the extrapersonal space (i.e., “how to go there”), within the distance up to which binocular depth perception operates (Palmisano et al. 2010), that is, a few meters in humans. Behavioral and modeling evidence indicate that this distance is of about 4 m (Fajen and Warren 2003), which is where the motor adjustments related to an avoidable obstacle begin. These adjustment skills develop during infancy with the acquisition of the capacity to anticipatorily plan an entire locomotor action (Rosenbaum 2009; Cowie et al. 2010).

### Multisensory environmental affordances for locomotor actions

Locomotor actions differ from other bodily actions, such as manipulative and defensive ones, particularly in their temporal unfolding. Indeed, locomotor actions frequently require variably long sequences of rhythmic muscular activity to bring some body part into contact with a target out of immediate reach. Hence, locomotor actions (such as jumping, running, or diving) can be planned when the subject identifies a potential target to be reached starting from a stationary condition or even during ongoing locomotion (e.g., while running, swimming or climbing). Concerning distinct sensory features of the environment, there are generally at least two types of signals, which can be identified as essential for planning locomotor actions in both static and dynamic conditions: (1) the location of the goal (Philbeck et al. 1997) and (2) the 3D structure and the nature of the medium (liquid, solid, slippery, etc.) in which locomotion will take place. As previously discussed for objects, distance estimation and the likelihood that the subject will attain the goal depend considerably on pragmatic information relevant to action planning and contextual elements (Proffitt et al. 2003; Stefanucci et al. 2005), thus supporting the idea that affordance competition can apply to distant targets as well.

**Fig. 4:**
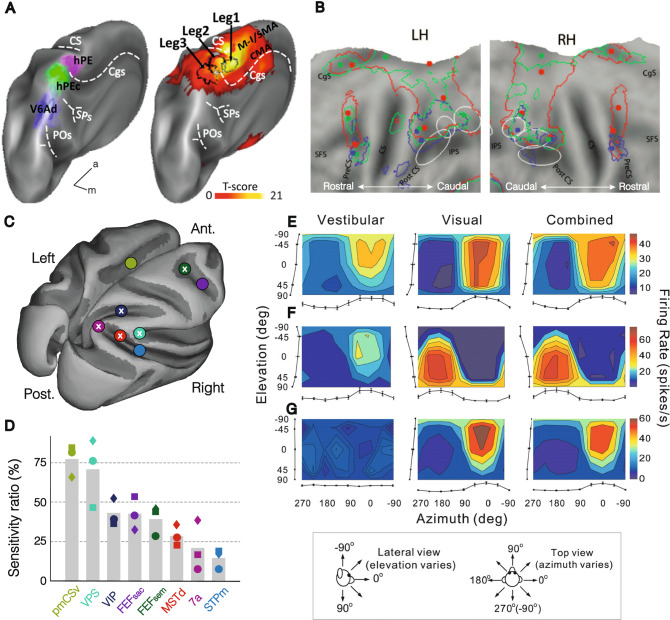
PPC regions involved in coding locomotor actions, visuo-vestibular integration, and optic flow. **A** Identification of the putative human PEc homolog via somatotopy (leg3, reproduced with permission from Pitzalis et al. 2019).** B** Human activation maps during observation of climbing (red line), locomotion (green line), and manipulation (blue line) (reproduced with permission from Abdollahi et al. 2013); the ellipses indicate, from rostral to caudal, the phAIP, dorsal intraparietal sulcus anterior (DIPSA), and medial (DIPSM) regions (the phAIP and the ventral part of the DIPSA correspond to the monkey AIP, dorsal DIPSA to VIP and DIPSM to rostral LIP). **C–D** Anatomical location **C** and sensitivity **D** of macaque cortical regions encoding the optic flow; crosses in panel C identify regions with visuo-vestibular convergence; circles, squares, and diamonds in panel D represent individual data for three different monkeys (modified with permission from Cottereau et al. 2017). **E**–**G** Visuo-vestibular integration in MSTd neurons. **E** Congruent; **F** opposite; **G** purely visual neurons (reproduced with permission from Gu et al. 2006). *CgS* cingulate sulcus, *SFS* superior frontal sulcus, *PreCS* precentral sulcus, *CS* central sulcus, *PostCS* postcentral sulcus, *IPS* intraparietal sulcus

The available evidence strongly indicates that the monkey parietal area PEc plays a crucial role in the integration of signals defining the location of the goal in terms of distance (Hadjidimitrakis et al. 2015) for locomotor action planning, and related to optic flow (Raffi et al. 2011) for obstacles avoidance. Area PEc is a visuo-somatosensory area located in the crown of the hemispheres between area PE, which is mainly somatosensory (De Vitis et al. 2019), and areas V6Ad and V6Av, which are mainly visual (Gamberini et al. 2020; Hadjidimitrakis et al. 2020). It exhibits a somatotopic organization that has a clear leg, in addition to arm, representation (Gamberini et al. 2020). Furthermore, its human homolog was identified (Pitzalis et al. 2019) in the crown of the hemisphere, in between the putative homologs of areas PE and V6A (Fig. 4A). This region corresponds to the third somatosensory leg representation in the parietal cortex, in addition to those of S1 and area PE, with the additional property of responding to the optic flow that is typically produced during the subject’s own locomotion. A survey of 237 PEc neurons revealed that 40% of them respond to complex visual stimuli (Gamberini et al. 2018), exhibiting typically large (30° × 30°) RFs that densely cover the visual field, thereby allowing population coding of the location of visual stimuli. These properties likely derive from afferents to area PEc from the medial parietal cortex (Bakola et al. 2010), encompassing the posterior cingulate cortex, area 7m and retrosplenial cortex, which encode particular locations along the subject’s route (Sato et al. 2006) because their place selectivity depends on the starting and final point (Sato et al. 2010). Furthermore, the human medial PPC has been shown to update the position of objects during self-motion (Wolbers et al. 2008), an important feature for signals involved in locomotor planning. The investigation of the neural mechanisms for encoding the location of an extrapersonal target to be reached by resorting to locomotion implies the use of wireless recording techniques (Berger et al. 2020). These techniques are rapidly spreading and may pave the way to novel, more integrated and ecologically valid approaches to the study of affordance competition among a larger variety of motor actions, including locomotor ones, when the potential target is located far from a freely moving subject.

Far less is known about the second visual signal, which concerns the 3D structure and the nature of the medium in which locomotion occurs. It is known that visually guided navigation relies largely on signals from the peripheral part of the visual field, even when rapid and adaptive navigation of obstacles is required, and that this ability is achieved during development (Franchak and Adolph 2010), but its neural bases have yet to be characterized with precision. Binocular mechanisms provide crucial information about the 3D layout of the environment, which is necessary for planning all types of visually guided (rhythmic and nonrhythmic) locomotor actions. For example, area PIP and CIP encode 3D surface orientation from disparity and project to area V6A (Van Dromme et al. 2016), thereby indirectly reaching area PEc, which seems to play the most important role in visually guided locomotion. Monocular information about the layout of the environment is provided by optic flow signals; these signals consist in the distribution of velocity vectors on the retinal array, which is typically induced by the relative motion of the observer with respect to the environment (Koenderink 1986). Optic flow provides information about (1) the object’s motion, even when the observer is moving, (2) the self-motion of the observer (see below), and (3) the 3D structure of the environment. The third aspect is processed in the MT complex of both human (Orban et al. 1999) and nonhuman (Xiao et al. 1997; Mysore et al. 2010) primates. In addition, comparative data indicate that 3D structure-from-motion signals are much more prevalent in the PPC of humans than in that of monkeys (Vanduffel et al. 2002), probably reflecting the change in lifestyle of hominins who came down from the trees and, as bipedal hunters and gatherers, moved long distances over ground planes. This parietal “scenic” component of 3D-structure-from-motion—as opposed to 3D-object-structure-from-motion signals processed in the MT complex—may be extracted in area V6 (Rosa and Tweedale 2001; Pitzalis et al. 2013; Fan et al. 2015), which processes large surfaces in peripheral vision (Galletti et al. 1999). Area V6 encodes, for example, information about the orientation and curvature of the ground when walking or of rocks’ or trunks’ surfaces during climbing, which may be further processed in the SPL, eventually reaching area PEc. Area PEc neurons have been shown to respond strongly to radial optic flow (Raffi et al. 2002, 2011), but it has yet to be investigated whether they can also analyze the speed distributions imposed onto these directional patterns to retrieve the 3D structure of the supporting surfaces during self-motion and to integrate peripheral details about obstacle location.

### Observing other’s locomotor actions and social affordances

Extending the social affordance hypothesis to locomotor actions implies that the observed actions of others can automatically trigger the neural representation of the subject’s own locomotor actions. Testing this hypothesis directly is technically difficult, because—as discussed above regarding environmental affordances—doing so would make it necessary to record neuronal activity in unconstrained situations, in which subjects could actually move toward or away from another subject. Nonetheless, preliminary evidence supports this possibility and indicates that it is as a suitable subject for future studies.

Indeed, human experiments have shown that subjects judge closer to them a target object in their extrapersonal space if it can be referenced to a real agent free to move toward it (Fini et al. 2015): if one accepts the hypothesis that space representations in a pragmatic format are based on the activation of the subject’s own potential motor plans, this finding can be plausibly interpreted as indicating that the subject’s own motor representations of walking actions are more readily accessible and enhanced when the observed object is potentially reachable by someone else. In line with this view, EEG experiments in humans have indicated that the observation of point light walkers on a screen simulating goal-directed locomotion recruits a component arising from the sensorimotor regions that correspond to the trunk and lower legs, but only when the point light walking configuration is preserved (Inuggi et al. 2018). Furthermore, fMRI studies have reported activation during the observation of two different locomotor actions (walking and climbing) in a common territory of the rostral part of the human SPL, corresponding to the phPEc (Abdollahi et al. 2013) (Fig. 4B), with additional evidence of mutual activations when participants observed or performed walking while being scanned, thanks to a rolling cylinder located just outside the scanner and enabling the testing of active walking (Dalla Volta et al. 2015). These findings support the idea that the observed locomotor actions of others can activate the same visuomotor parietal regions that support the planning of the subject’s own locomotion. Nonetheless, the complexity of locomotor behavior is associated with the lack of single-neuron data supporting self/other specificity in their neural representation, which should be the focus of future studies (e.g., by investigating the selectivity for different locomotor-action exemplars in area PEc of stationary subjects).

Locomotor actions are aimed primarily at bringing the body closer to physical objects or other subjects, and their visual-to-motor mapping may be even broader than previously shown for manipulative actions. Thus, it can be predicted that a variety of observed actions performed by others, but not necessarily belonging to the locomotor-action class could afford locomotor-action plans in the observer’s brain. For example, athletes exhibit a remarkable ability to predict and anticipates others’ manual actions based on extremely subtle kinematic details, such as the little-finger angle during a basketball shot (Aglioti et al. 2008); such a detection can readily afford locomotor actions to take possession of the ball. Similar evidence has been provided for foot actions, such as kicking that can afford appropriate parrying behavior in expert players (Tomeo et al. 2013; Makris and Urgesi 2015) and likely extend to a variety of contextual situations (e.g., exploratory behavior, manipulation of food items, interaction with a social partner, etc.) in human and nonhuman primates’ daily life.

### Multisensory and motor signals about executed locomotor actions

Locomotor actions rely heavily on signals caused by the unfolding of the subject’s own action. These signals are encoded with reference to different coordinate systems, such as the eye (eye centered), the head (head centered), or the body (body or word centered) (Chen et al. 2013). Clearly, proprioceptive and visual signals concerning the subject’s own body movements and the possible perturbation offered by obstacles in the environments play a key role in the online control of locomotion (Frost et al. 2015; Kim et al. 2018), but this is beyond the scope of this article. Nonetheless, there are at least two additional important sources of input that are critical for goal-directed, visually guided locomotion: (1) optic flow deriving from one’s own locomotion (already mentioned above), including visual heading, and (2) vestibular signals.

Visual heading signals specify the direction of self-motion in space derived from the analysis of the location of expansion/contraction focus in the radial component of optic flow. The first evidence of neurons encoding expansion and contraction was reported in MSTd (Saito et al. 1986), but similar heading signals have been reported subsequently in many additional areas, such as VIP (Schaafsma and Duysens 1996) and area 7a (Siegel and Read 1997). For example, an fMRI study in the alert monkey (Cottereau et al. 2017) comparing global flow (including a single focus of expansion, compatible with egomotion) with composite flow (including multiple foci of expansion, incompatible with egomotion) to isolate heading signals confirmed the involvement in heading processing of a set of regions (Fig. 4C–D) in which single-neuron evidence of heading coding were reported, such as the frontal eye field (FEF), the visual posterior sylvian (VPS) and the STPm (Andersen et al. 1985; Steinmetz et al. 1987; Raffi et al. 2002, 2011; Chen et al. 2013; Fan et al. 2015; Gu et al. 2016), in addition to MSTd, VIP and area 7a, as mentioned above. In all the extant studies, the monkeys passively viewed the stimuli, but neuronal responses recorded while they were discriminating heading direction revealed that it is encoded in MSTd, VIP and parieto-insular vestibular cortex (PIVC), but only the two latter areas also specify the monkey’s active choice during perceptual discrimination (Gu et al. 2007; Chen et al. 2013, 2021).

The vestibular apparatus provides another important signal for goal-directed locomotion. Studies in which the monkey was moved passively in a sled along the three axes of space have shown that areas MSTd (Gu et al. 2006), VIP (Chen et al. 2011a), VPS (Chen et al. 2011b), FEFsem (Gu et al. 2016) and 7a (Avila et al. 2019) process vestibular in addition to visual heading cues. Notably, the visual and vestibular signals are integrated in most of these areas, giving rise to congruent (similar selectivity in the elevation-azimuth plane) and opposite (dissimilar selectivity in the elevation-azimuth plane) visuo-vestibular neurons (Fig. 4E–G), with the exception of area 7a (Avila et al 2019). Area 7a, which is probably more involved in navigation than locomotion, also stands out for exhibiting its own vestibular input directly from the vestibular thalamus (Faugier-Grimaud and Ventre 1989). The extensive integration of visual and vestibular signals is also prevalent in natural settings, and PIVC/VPS, but not the VIP, play a causal role in heading discrimination in ethological conditions (Chen et al. 2016). PIVC/VPS has direct connections with area PEc (Bakola et al 2010) and likely represents the main source of ethologically valid heading signals to the latter area.

### Summing up: multimodal signals for locomotor-action planning

**Fig. 5:**
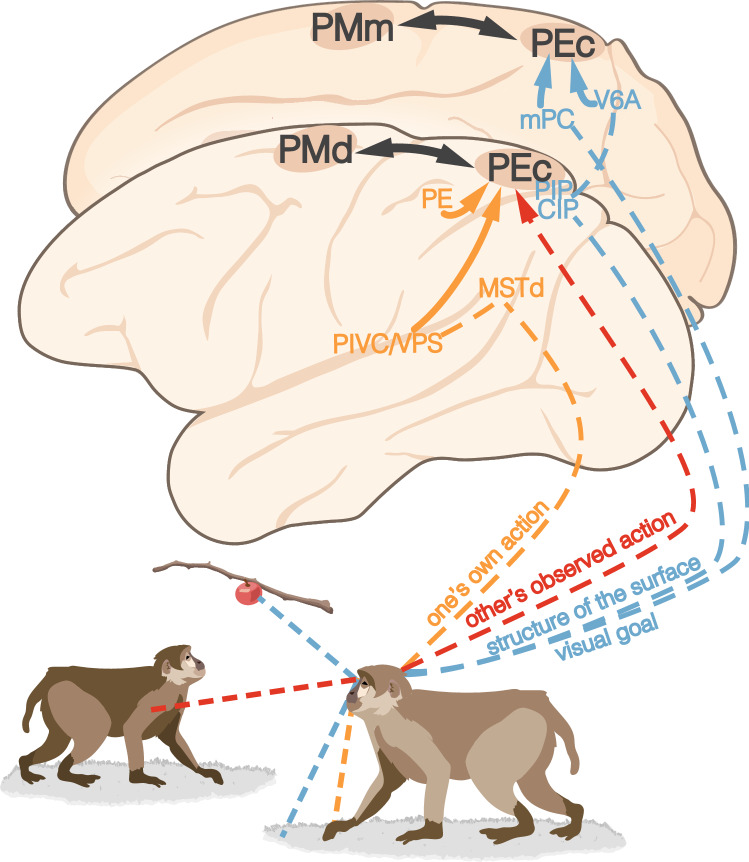
Signals and parieto-frontal circuits for locomotor actions. Convergence of visual information about different aspects of the environment (blue), including visual goals and structure of the surface or medium through which locomotion occurs, others’ observed actions (red), and visual/proprioceptive signals deriving from the subject’s locomotion (orange) in area PEc, the PPC territory devoted to locomotor actions. Note that evidence concerning the steps in the processing of others’ locomotor actions is still too scarce to be explicitly represented

The locomotor action class further extends the model proposed for manipulative and defensive actions (Fig. 5): physical and social objects that populate our environment and cannot be reached by a subject that remains still constitute potential targets of locomotor actions. Thus, physical and social affordances may activate locomotor actions as well, making their direct investigation particularly challenging, because it requires not only untethered recording systems, but also the exploration of larger and more variable locomotor spaces with a variety of bodily actions in 3D. Pioneering studies with smaller animal models, such as bats (Sarel et al. 2017) and rodents (Kingsbury and Hong 2020), are opening intriguing and almost completely unexplored research fields for nonhuman primate research as well (Mao et al. 2020), which promise to shed light on highly complex and rarely investigated human behaviors in close-to-natural situations.

## From bodily actions to artificial implements

The main focus of this review is on the visual signal for bodily actions of primates, and this entails at least briefly considering actions performed with the aid of man-made objects and tools. Indeed, tool use arose in the human lineage almost 3 million years ago (Harmand et al. 2015), and it appears to varying extents in many nonhuman primate species (Macellini et al. 2012; Falótico et al. 2019; Manrique et al. 2021), making it an ancient part of our evolutionary history (Haslam et al. 2017). Any object can be considered a tool if its use allows one to alter the position, shape, or condition of another object; tools thus range from very simple objects available in the environment, such as stones used by shellfish foraging monkeys (Luncz et al. 2017), to artificial implements designed and built exclusively by humans to overcome the limitations of actions performed only with our biological effectors and to be more powerful or precise, to reach unreachable places, or to act over longer distances. Artifacts, such as screwdrivers, pliers, umbrellas, bicycles, or cars, can be considered as an amplification of the goals (e.g., manipulation, defense, locomotion, etc.) that the various bodily action classes allow one to achieve. The most notable effect of these man-made artifacts is their capacity to modify and fine-tune environmental affordances; at the same time, man-made artifacts require the presence of someone who teaches and someone who learns how to purposefully and appropriately use them to achieve the desired goal. Thus, artifacts entail the flexible integration of object and social affordances; this integration stems directly from the potential relationship between an observer, on the one side, and an object alone or an object with another agent, on the other side, in a highly complex, socially driven context that must crucially include the learning history of the observer (Ramsey et al. 2021). Here, we will briefly discuss the use of tools and implements as an extension of bodily actions (a discussion of the invention or creation of new tools or implements is beyond the scope of the present review).

Current neuroscientific evidence indicates that, despite the widely shared use of tools in primates, only the human brain has evolved a parietal region dedicated to planning the use of tools: the left anterior supramarginal gyrus (aSMG), adjacent to phAIP (Peeters et al. 2009; Brandi et al. 2014; Caruana et al. 2017). The close proximity and link between these two regions suggest that when an object can be used as tool, its affordances are twofold: first, phAIP can code the bodily actions that enable the subject to interact with the tool as an object; second, the aSMG can code the specific sequence of actions with which the agent can exploit the object as a tool (Orban and Caruana 2014) that expand the set of motor goals the agent can achieve when using it (Maravita and Iriki 2004).

Affordances related to tool objects can also be evoked simply by seeing others using a given tool; thus, just as OMAs represent a source of social affordances in phAIP, the aSMG becomes active during tool action observation (Peeters et al. 2009) and may therefore constitute an additional source of social affordances in humans. Indeed, as discussed above for manual actions, tool action can automatically index a variety of potential motor actions (with or without the aid of a tool), thereby offering the observer a large set of potential bodily actions from which one can be selected and possibly performed in response to the action performed by another individual. Of course, the signals and pathways conveying information about the sensory features of the environment described above for other action classes may still be valid when dealing with artifacts and tool use, because they extends the domain of motor possibilities already associated with different classes of bodily actions.

## Conclusions

A recently proposed model of the planning of manipulative actions posits that three main types of signals enable the planning and selection of goal-directed manipulative actions, namely, (1) objects’ physical properties, (2) others’ observed actions, and (3) own action-related signals, thereby extending the concept of affordance from viewed objects to observed actions (Orban et al. 2021). Here, we reviewed an extensive body of evidence that supports the possibility of extending this model to other action classes, focusing on defensive and locomotor actions but also offering a brief discussion of actions performed with tools. We showed that the same type of signal can be differently modulated and variably shared by objects and social-affordance properties within each action domain but, for several of these domains we also stressed the limits of our present knowledge, especially for action classes requiring unconstrained, free movement of the subject in the environment. Another set of experiments, which might be more easily performed even in constrained monkeys, relates to the single-neuron selectivity for observed-action exemplars of the defensive and locomotor-action classes, because most of the extant literature has focused on manual actions or, more specifically, grasping. In fact, a great deal of work on defensive and locomotor actions remains to be done, and novel approaches and technologies that allow us to investigate brain activity in freely moving nonhuman primates with more ecologically valid stimuli will likely be crucial.

Studies in different animal models have supported the idea that maps for actions of self and others exist in the parietal cortex of rodents as well (Mimica et al. 2018; Tombaz et al. 2020; Ebbesen and Froemke 2021), suggesting that such an exploitation of visual signals for action planning (Ukezono and Takano 2021) is an ancient evolutionary achievement that is widespread among mammals. Furthermore, studies in mice and bats have provided interesting evidence of the cellular mechanisms that may help agents to exploit social information to plan their behavioral responses (Kingsbury and Hong 2020), thereby directly supporting the social-affordance hypothesis. It should also be noted that the prominent role of noncortical brain structures, such as the basal ganglia and cerebellum, in other vertebrates that are capable of social learning and of coordinating their behaviors with those of others (Bonini and Ferrari 2011), suggests that these regions may have played a fundamental role in the evolution of the cortical circuits reviewed here. Indeed, studies with fMRI (Errante and Fogassi 2020) and intracranial recordings (Alegre et al. 2010) support the involvement of these (Caligiore et al. 2013) and other (Sinke et al. 2010) noncortical brain regions in the encoding of observed and executed actions, emphasizing the need to clarify the underlying cellular mechanisms.

These future steps will play a crucial role in empirically validating the proposed model and in clarifying the organization and functioning of parietal maps and of parieto-frontal circuits for the processing of physical and social sensory signals necessary to the planning of one’s own bodily actions.

## Data Availability

Not applicable.
